# Reliability of an ultrasound imaging acquisition procedure for examining osteoarthritis in the first metatarsophalangeal joint

**DOI:** 10.1002/jfa2.12002

**Published:** 2024-03-29

**Authors:** Prue Molyneux, Catherine Bowen, Richard Ellis, Keith Rome, Kate Fitzgerald, Phillip Clark, Matthew Carroll

**Affiliations:** ^1^ School of Clinical Sciences Auckland University of Technology Auckland New Zealand; ^2^ Active Living and Rehabilitation: Aotearoa New Zealand Health and Rehabilitation Research Institute School of Clinical Sciences Auckland University of Technology Auckland New Zealand; ^3^ School of Health Sciences Faculty of Environmental and Life Sciences University of Southampton Southampton UK; ^4^ Centre for Sport, Exercise and Osteoarthritis Versus Arthritis University of Southampton Southampton UK; ^5^ Beyond Radiology Auckland New Zealand

**Keywords:** foot, metatarsophalangeal joint, osteoarthritis, ultrasonography

## Abstract

**Objective:**

Given the ability of ultrasound imaging (USI) to depict tissue‐specific morphological changes before the onset of pain and before the point of irreversible structural damage, USI could play a fundamental role in earlier detection and assessment of foot osteoarthritis (OA). The current guidelines require further refinement of anatomical landmarks to establish a standardized imaging procedure to improve the interpretability and reproducibility between studies evaluating the first metatarsophalangeal joint (MTPJ). The aims were to develop an USI acquisition procedure and grading system to examine OA features in the first MTPJ and to determine intra‐examiner and inter‐examiner reliability of a newly developed USI acquisition procedure.

**Design:**

Thirty participants with first MTPJ OA confirmed radiographically with the use of the La Trobe Foot Atlas were included. An experienced sonographer applied a newly developed USI procedure to examine the following features: joint effusion, synovial hypertrophy, synovitis, joint space narrowing, osteophytes, and cartilage thickness. A semiquantitative grading system was applied to all features. A continuous measure was also examined for osteophyte size, joint space narrowing, and cartilage thickness. To determine the intra‐examiner and inter‐examiner reliability, an experienced radiologist and sonographer applied the developed grading system to the images acquired from two imaging sessions. Intra‐examiner and inter‐examiner reliability were calculated using intraclass correlation coefficients (ICCs).

**Results:**

ICCs for intra‐examiner between session reliability ranged from 0.58 to 0.92 for semiquantitative grading and 0.39 to 0.94 for continuous measures. Joint effusion and osteophytes achieved the highest intra‐examiner reliability (ICC = 0.78–0.94). ICCs for session one inter‐examiner reliability ranged from 0.61 to 1.0 for semiquantitative grading; all continuous measures had an ICC of 1. ICCs for session two inter‐examiner reliability ranged from 0.55 to 1.0 for semiquantitative grading and 0.9 to 0.97 for continuous measures. Inter‐examiner reliability was good for grading joint effusion (ICC = 0.55–0.62) and was excellent for all other USI features (ICC = 0.77–1.0).

**Conclusion:**

The USI acquisition procedure and grading system are reliable in evaluating first MTPJ OA features in participants with radiologically confirmed OA. The study will inform the methodological development of an ultrasound atlas for grading the degree of osteoarthritic change in the first MTPJ.

**Abbreviations**
EULAR: European League Against Rheumatism
ICCs: intraclass correlation coefficients
LFA: La Trobe Foot Atlas
MTPJ: metatarsophalangeal joint
OA: osteoarthritis
OMERACT: Outcome Measures in Rheumatology
USI: ultrasound imaging

## 1 INTRODUCTION

Ultrasound imaging (USI) is the most rapidly developing technique in musculoskeletal imaging, with continuing technological advances broadening its application [1]. USI potentially affords inherent advantages for the diagnosis of osteoarthritis (OA) due to its ability to detect inflammatory joint pathology that is otherwise not detected by clinical examination [2, 3], and can reliably quantify both bone and soft‐tissue abnormalities [4]. Given the ability of USI to detect tissue‐specific morphological changes before the onset of pain and before the point of irreversible structural damage, USI may play a fundamental role in the early detection and assessment of OA [5, 6]. However, the role of USI for OA diagnosis in foot joints, such as the first metatarsophalangeal joint (MTPJ), has not been clearly defined.

The foot is a target region for OA [7], yet foot research is a relatively nascent and evolving discipline within the broader field of OA [8, 9]. Within the foot, USI has been shown to significantly increase diagnostic confidence in differentiating OA from other inflammatory arthritis [10]. However, current USI grading systems and atlases applied to OA have been largely extrapolated from recommendations originally developed for populations with rheumatoid arthritis (RA) [11]. Given that inflammation associated with OA is fundamentally different from that in RA [12, 13, 14, 15, 16, 17], the validity of using definitions, grading systems, and atlases originally developed for RA must be considered. This reinforces the need for validated OA‐specific grading systems that depict the disease progression and may be more helpful in elucidating the role of inflammation in foot OA.

In our recent systematic review, we identified inconsistencies in the assessment of USI features, the definition of features, and the grading systems used to determine the degree of OA change in peripheral joints [11]. Inconsistencies were identified by our scoping review against international guidelines and limited implementation of consensus‐based recommendations for USI procedure guidance when evaluating the first MTPJ [18]. It is currently unclear which USI procedure should be used to examine the first MTPJ OA. Current guidelines require further refinement of anatomical landmarks to establish a standardized imaging procedure to improve interpretability and reproducibility between studies that evaluate the first MTPJ. To address these research gaps, we conducted a Delphi exercise to gain consensus concerning which USI features should be assessed and graded, and what USI procedure should be performed when examining the first MTPJ OA. The Delphi study identified the essential components that the USI acquisition procedure should encompass when examining the first MTPJ OA [19].

The ability to reliably quantify the degree of structural and inflammatory change in the first MTPJ OA will provide a more sensitive method for assessing disease severity. Therefore, the study aimed to (1) develop an USI acquisition procedure and grading system to examine OA features in the first MTPJ, and (2) determine the intra‐examiner and inter‐examiner reliability of the newly developed USI acquisition procedure.

## 2 METHODS

### 2.1 Study design

The USI acquisition procedure was developed using an evidence‐based approach with findings from a systematic review [11], a scoping review [18], and a Delphi consensus study [19] serving as a basis for development. The feasibility work and the final refinement of the USI acquisition procedure were determined by an experienced sonographer (KF), radiologist (PC) and a podiatrist (PM). The examiners (PM, KF, and PC) piloted and clarified the USI examination methodology prior to participant recruitment. The purpose of the process was to establish a shared understanding and agreement regarding the USI acquisition procedures, image interpretation, and grading. A participant‐based exercise was used to determine the reliability of the newly developed USI acquisition procedure and grading system for evaluating osteoarthritic change in the first MTPJ. The study details were reported in accordance with the European League Against Rheumatism Recommendation Checklist for Reporting of Rheumatic and Musculoskeletal USI Research (Supporting Information S1) [20]. The study was approved by the Southern Health and Disability Ethics Committee, HDEC Ethics Reference: 2022 FULL 12721.

#### 2.1.1 Participants

**FIGURE 1:**
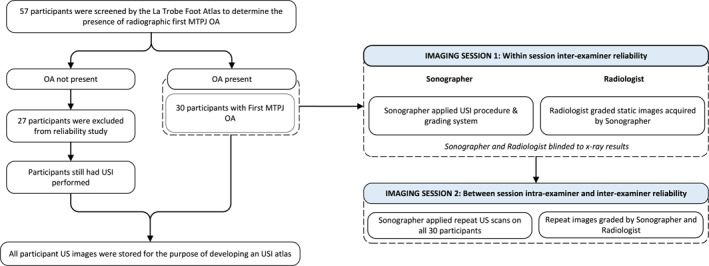
Overview of the participant journey.

Participants with suspected or previously diagnosed first MTPJ OA, and over 20 years of age were recruited from the general population in Auckland, New Zealand. Participants were recruited through professional interactive networks, social media (Twitter and Facebook), and local newspaper advertisements. Exclusion criteria were the possibility of pregnancy, the presence of any other inflammatory musculoskeletal condition, history of a first MTPJ surgery or foot and/or ankle surgery in the last 3 months. To ascertain the reliability of the newly developed USI procedure, the La Trobe Foot Atlas (LFA) was used to screen study participants by determining the presence of radiographic first MTPJ OA [21]. Fifty‐seven participants were screened in the first imaging session, of which 30 participants had radiographically confirmed first MTPJ OA and returned for a repeat USI examination in the second imaging session. Each participant was randomly assigned an alphanumeric code upon entry into the study. Written informed consent was obtained from all patients before the study. Figure 1 provides a graphical overview of the participant journey.

### 2.2 Imaging session one

Imaging session one involved a demographic, radiographic and sonographic assessment, all performed at a private medical imaging facility. All assessments were conducted sequentially within a 60‐min session in three separate rooms. First, demographic data were obtained for each participant (age, gender, height, weight, body mass index, ethnicity, and first MTPJ affected). Second, an X‐ray was taken to determine the presence of radiographic first MTPJ OA. Third, participants underwent an USI examination using our newly developed USI acquisition procedure and grading system.

#### 2.2.1 Radiographic assessment and screening

To determine eligibility for the study, dorsal/plantar and lateral weight bearing radiographs were obtained by an experienced radiographer. A radiologist (PC) used the LFA to determine the presence of radiographic first MTPJ OA and to screen participants into the study [21]. The LFA considers OA to be present when a score of 2 or greater for osteophytes or joint space narrowing is documented from either the dorsal/plantar or lateral view [21]. One radiologist (PC) assessed and reported on all radiographs.

#### 2.2.2 USI assessment

Directly after the X‐ray examination all 57 participants received an USI examination, regardless of the X‐ray result. Only the 30 participants that had confirmed radiographic first MTPJ OA ultrasound images were invited to return for a repeat USI examination in imaging session two. Ultrasound images from all 57 participants were stored for the purpose of developing a USI atlas as part of a future study. However, the 27 non‐OA participants were excluded from the reliability study and could not proceed into the second imaging session.

**FIGURE 2:**
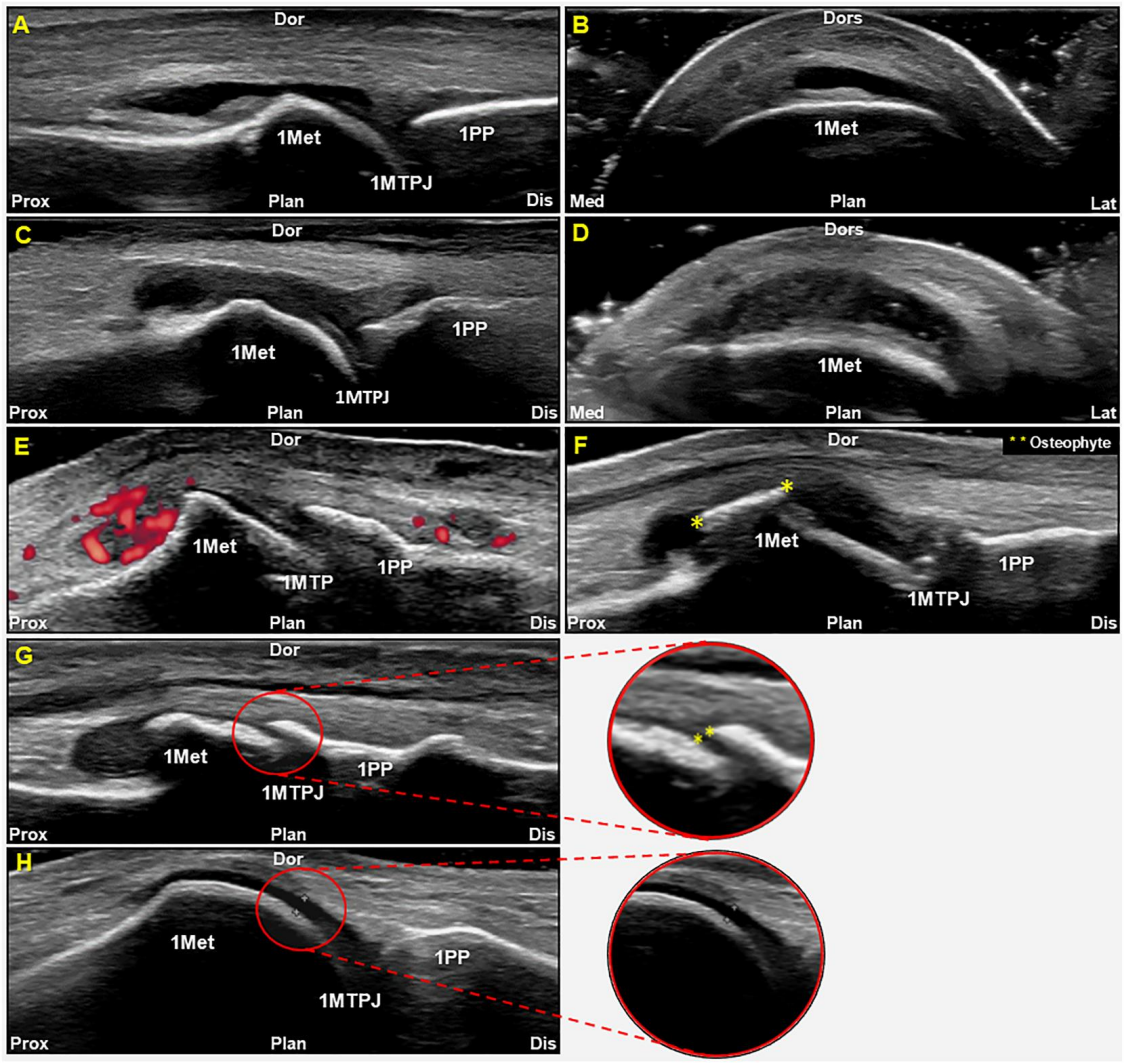
Ultrasound imaging first MTPJ osteoarthritis features that were examined. (A) Joint effusion in longitudinal; (B) joint effusion in transverse; (C) synovial hypertrophy in longitudinal; (D) synovial hypertrophy in transverse; (E) synovitis; (F) osteophyte; (G) joint space narrowing; (H) cartilage thickness. 1Met, first metatarsal; 1MTPJ, first metatarsophalangeal joint; 1PP, first proximal phalanx; Dis, distal; Dor, dorsal; Lat, lateral; Med, medial; Plan, plantar; Prox, proximal.

The USI acquisition procedure and grading system examined the following features of the first MTPJ: joint effusion, synovial hypertrophy, synovitis, joint space narrowing, osteophytes, and cartilage thickness (Figure 2). A semiquantitative grading system was applied to all features (0 = absent, 1 = mild, 2 = moderate, 3 = severe) (Supporting Information S2). To mitigate problems with adequate discrimination between intermediary grades of cartilage thickness, a 0–2 semiquantitative grading system was applied. This is consistent with a recent hand OA study [22]. A continuous measure was also examined for osteophytes (mm), joint space narrowing (mm), and cartilage thickness (mm). For the examination of osteophytes, the first proximal phalanx and the first MTPJ head were evaluated as a whole, with the largest osteophyte independently defining the score (Figure 2F). The joint space was measured at the largest joint space from the distal bony edge of the first metatarsal to the most proximal bony edge of the first proximal phalanx (Figure 2G). Cartilage thickness was measured at the sharpest margin of the articular cartilage from the articulating edge (Figure 2H).

**FIGURE 3:**
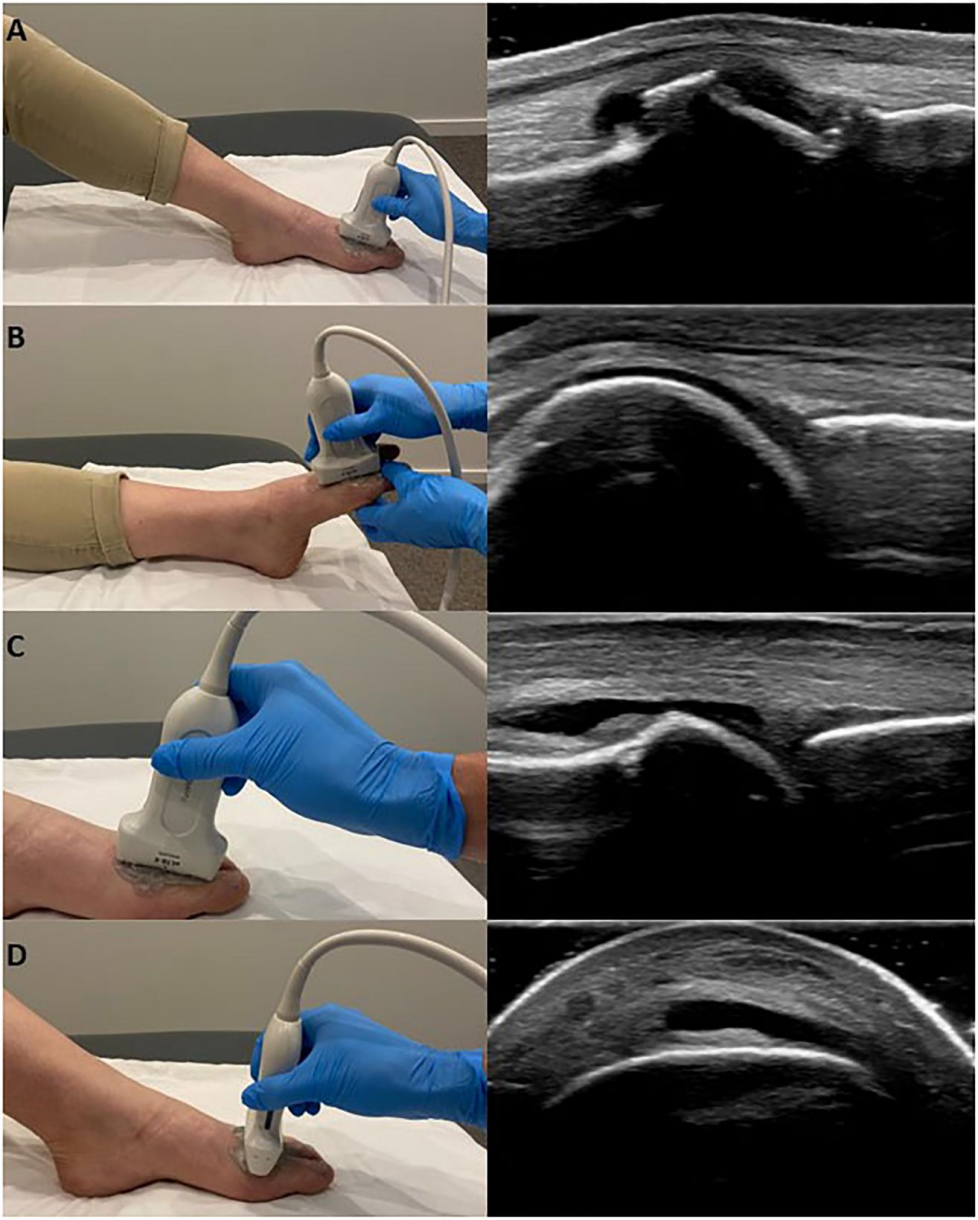
Participant and probe positioning for the ultrasound imaging acquisition procedure. (A) Participant positioning for assessing all features; (B) participant positioning for assessing cartilage; (C) probe positioning for longitudinal scans; (D) probe positioning for transverse scans.

Participant and probe positioning was informed through the findings of our Delphi study [19]. All features were assessed with the participant positioned supine with the knee flexed, the foot flat on the plinth, and the first MTPJ in neutral (Figure 3A). The cartilage was examined with the knee extended, ankle plantarflexed, and the first MTPJ positioned in neutral then moved through plantarflexion during scanning (Figure 3B). Positioning the first MTPJ in plantarflexion opened the joint space, optimizing the image for examining cartilage thickness. All USI features were assessed in the dorsal view with the probe positioned longitudinally; a transverse orientation was also applied to examine joint effusion, synovial hypertrophy, and osteophytes. For longitudinal scans the probe was positioned on the dorsal aspect of the forefoot, parallel to the first metatarsal head and proximal phalanx, with the joint line central to the image (Figure 3C). For transverse scans the probe was positioned on the dorsal aspect of the foot, perpendicular to the diaphysis of the first metatarsal then moved distally to the diaphysis of the first proximal phalanx, with the joint line central to the image (Figure 3D).

A sonographer (KF) with more than 15 years of experience in musculoskeletal USI applied the newly developed USI acquisition procedure and grading system to evaluate the first MTPJ OA. Grading of each USI feature was performed immediately after the imaging session. To determine inter‐examiner reliability, a radiologist (PC) with more than 20 years' experience applied the developed grading system to the static images acquired by the sonographer. Both examiners were blinded to the radiographic results, clinical data, and each other's gradings.

#### 2.2.3 Equipment

A Philips Epiq Elite HW B.2 ultrasound machine, equipped with a multifrequency linear transducer (eL18–4 MHz) (©2015 Koninklijke Philips N.V.), was used to acquire images of the first MTPJ. The USI device did not change during the session or between sessions. Gray scale was used to examine all features (128 Hz Gray Map 3, greyscale gain 30%–40%, dynamic range 74%–68%, Med, 2D Opt Res, SonoCT, XRES 4) and power Doppler was applied for the examination of synovitis (PRF 700 Hz, Non‐directional Flow Color Map CPA 3, Color gain 50%–65%, Wall Filter 63 Hz, Frequency 6.2 MHz). At the beginning of each scanning session focus was positioned at the level of the region of interest. Color gain was adjusted below the degree that caused the appearance of noise artifacts.

### 2.3 Imaging session two: Repeat USI

To determine intra‐examiner between session reliability, 30 participants with radiographically confirmed the first MTPJ OA attended a second USI session for a repeat USI assessment of the same first MTPJ within two weeks of their first USI assessment. The minimum time between USI sessions was 1 week. The sonographer from session one applied the same USI procedure and grading system. To determine inter‐examiner reliability for session two, the radiologist from session one applied the developed grading system to static images acquired from session two. Both examiners were blinded to session one and each other's gradings.

### 2.4 Statistical analysis

Statistical analysis was performed using SPSS V.28 (SPSS Inc.). Descriptive statistics of categorical data are presented as frequencies and percentages, while means and standard deviations (SDs) were calculated for descriptive statistics of continuous variables. Intraclass correlation coefficients (ICCs) and 95% confidence intervals (CIs) using two‐way mixed model (3,1) with absolute agreement were calculated to examine the intra‐examiner between session reliability. ICCs and 95% CIs using two‐way random (2,1) with absolute agreement were calculated to examine the inter‐examiner reliability among the sonographer and radiologist for session one and two. The following criteria were applied, <0.40 signified poor reliability; 0.40–0.75 fair to good reliability; and >0.75 excellent reliability [23].

## 3 RESULTS

Table 1 details the demographic characteristics of the 30 participants (25 female, 5 male) included in the reliability study. Table 2 displays the intra‐examiner between‐session reliability. ICCs for intra‐examiner between session reliability ranged from 0.58 to 0.92 for semiquantitative grading and ranged from 0.39 to 0.94 for continuous measures. Joint effusion and osteophytes achieved the highest intra‐examiner reliability. Table 3 displays the inter‐examiner reliability of sessions one and two. ICCs for session one inter‐examiner reliability ranged from 0.61 to 1.0 for semiquantitative grading and all continuous measures had an ICC of 1. ICCs for session two inter‐examiner reliability ranged from 0.55 to 1.0 for semiquantitative grading and ranged from 0.9 to 0.97 for continuous measures. Inter‐examiner reliability was good for grading joint effusion and was excellent for all other USI features.

**TABLE 1:** Participant demographic data.

Number		30
Age, years, mean (SD)		54.4 (12.5)
Sex, *n* (%)	Male	5 (17%)
	Female	25 (83%)
Ethnicity, *n* (%)	NZ European	22 (73%)
	Māori	4 (14%)
	White British	2 (7%)
	Asian	1 (3%)
	Russian	1 (3%)
Body mass index, kg/m^2^, mean (SD)		29.8 (6.7)
First MTPJ affected	Left	13 (43%)
	Right	17 (57%)

Abbreviations: MTPJ, metatarsophalangeal joint; *n*, number; SD, standard deviation.

**TABLE 2:** Intra‐examiner between‐session reliability.

USI feature		ICC	95% CI
Joint effusion	Semiquantitative grade (L)	0.82	0.66–0.91
	Semiquantitative grade (T)	0.78	0.59–0.89
Synovial hypertrophy	Semiquantitative grade (L)	0.65	0.39–0.82
	Semiquantitative grade (T)	0.69	0.44–0.84
Synovitis	Semiquantitative grade	0.61	0.33–0.79
Joint space narrowing	Semiquantitative grade	0.58	0.29–0.78
	Continuous measure	0.67	0.42–0.83
Osteophytes	Semiquantitative grade	0.92	0.84–0.96
	Continuous measure	0.94	0.87–0.97
Cartilage	Semiquantitative grade	0.68	0.43–0.83
	Continuous measure	0.39	0.06–0.65

Abbreviations: CI, confidence interval; ICC, intraclass correlation coefficient; L, longitudinal plane; T, transverse plane; USI, ultrasound imaging.

**TABLE 3:** Inter‐examiner reliability of session 1 and 2.

USI feature		Session 1 ICC (95% CI)	Session 2 ICC (95% CI)
Joint effusion	Semiquantitative grade (L)	0.62 (0.34–0.80)	0.57 (0.27–0.77)
	Semiquantitative grade (T)	0.61 (0.32–0.79)	0.55 (0.22–0.75)
Synovial hypertrophy	Semiquantitative grade (L)	0.82 (0.66–0.91)	0.61 (0.33–0.80)
	Semiquantitative grade (T)	0.77 (0.58–0.88)	0.77 (0.57–0.88)
Synovitis	Semiquantitative grade	0.89 (0.78–0.95)	0.97 (0.93–0.98)
Joint space narrowing	Semiquantitative grade	1.0	1.0
	Continuous measure	1.0	1.0 (0.99–1.0)
Osteophytes	Semiquantitative grade	1.0	0.96 (0.92–0.98)
	Continuous measure	1.0	0.99 (0.99–1.0)
Cartilage	Semiquantitative grade	0.92 (0.84–0.96)	0.90 (0.80–0.95)
	Continuous measure	1.0 (1.0–1.0)	0.97 (0.93–0.98)

Abbreviations: CI, confidence interval; ICC, intraclass correlation coefficient; L, longitudinal plane; T, transverse plane; USI, ultrasound imaging.

## 4 DISCUSSION

The developed USI acquisition procedure and grading system are reliable in evaluating first MTPJ OA features in participants with radiologically confirmed OA. Data revealed the assessment of joint effusion, synovial hypertrophy, synovitis, joint space narrowing, osteophytes, and cartilage thickness had good to excellent intra‐examiner and inter‐examiner reliability. Poor intra‐examiner reliability was only reported for cartilage thickness when assessed as a continuous measure. Absolute agreements were excellent for osteophytes and joint space narrowing.

It is well understood that inflammation is an important driver of the disease and contributes to the structural progression of OA [13, 24, 25]. Despite this contemporary understanding, clinicians and researchers are currently confined to radiographic grading or grading originally designed for RA when examining foot OA. The distinct difference of inflammation experienced in OA compared to RA and the inability of conventional radiography to detect inflammatory features provides significant limitations. Therefore, the development of our USI grading system is a fundamental step in determining the role of inflammation for first MTPJ OA and the prognostic value for structural progression. Furthermore, it is generally accepted that radiological progression may not always correlate with pain, function and/or impact on activities of daily living. USI may further drive our understanding of factors which may be more important at patient level (i.e., health related quality of life). Therefore, USI may potentially drive more targeted and personalized interventions in the future.

It is a pivotal finding that our developed USI procedure and grading system reported good to excellent intra‐examiner and inter‐examiner reliability for all inflammatory OA features. Particularly, given the marked variations across studies in terms of how synovitis, synovial hypertrophy, and joint effusion are defined and categorized as USI features [11]. Consequently, it is essential to discriminate the existing disparities among various entities of synovial pathology that serve as indications of inflammation [11]. In line with the Outcome Measures in Rheumatology and OA study, we scored greyscale inflammatory abnormalities for synovial hypertrophy and joint effusion separately. Synovitis was examined as a separate entity by power Doppler signal (flow signal detected within synovial hypertrophy was considered a sign of synovitis) [26, 27]. Due to the marked variation in prevalence between grayscale and Doppler detected inflammatory features demonstrated in hand OA [26], only including greyscale features indicative of inflammation may result in OA being underestimated. A recent study that used magnetic resonance imaging to examine first MTPJ OA, was limited by the fact that they included effusion and synovitis as a combined proxy measure for synovitis, termed “effusion‐synovitis” [28]. Given the prognostic value of inflammatory features and the sensitivity USI possesses in detecting subclinical inflammatory change [2, 3], the inclusion of multiple inflammatory features that can be reliably quantified as separate entities, may be more helpful in elucidating the role of inflammation in OA.

The poor intra‐examiner reliability for examining cartilage as a continuous measure may be attributed to scoring cartilage based on a single thickness measurement. It may be that as cartilage may not be uniform across the entire joint surface, a singular measurement of thickness may not provide an accurate representation of cartilage damage across the whole joint surface. Therefore, the ability to consistently examine the exact same part of cartilage, between sessions, will influence the reliability of this measure. The technique we employed requires examiners to measure a vertical line with consistent perpendicular alignment between cartilage borders at a subjective location. Small deviations in the location and orientation can result in thickness differences and measurement variance between sessions and/or examiners [29]. This finding is consistent with previous OA and RA studies, which have reported difficulties when examining cartilage damage [22, 28]. Our poor intra‐examiner reliability results may also be attributable to practical difficulties associated with the scanning of cartilage. To obtain the exact same ultrasound image, the beam angle and location used in session one would need to be precisely replicated in session two. This may not have occurred for all repeat scans which may have influenced the cartilage measurement [30, 31]. Conversely the excellent inter‐examiner reliability for cartilage thickness as continuous measure between session one and two may be explained by the fact that the radiologist graded already acquired images of cartilage thickness.

A previous attempt to develop semiquantitative 0–3 grading for cartilage in hand OA found moderate intra‐reader and only fair inter‐reader agreement [32]. Even supporting definitions could not help to sufficiently discriminate between intermediary grades. A recent study on cartilage in RA patients simplified the scoring to a 0–2 scale and reported excellent intra‐reader and moderate inter‐reader reliability in the metacarpophalangeal joint [33]. Therefore, to mitigate issues with mid‐range subjective grading we opted for a 0–2 semiquantitative scoring system based on the morphological integrity of the superficial interface of the cartilage and the cartilage thickness. To aid in the visualization of cartilage thickness, the acquisition procedure was modified to include plantarflexion of the first MTPJ. Consequently, the degree of plantarflexion achieved between sessions may have varied, this would undoubtedly have influenced cartilage thickness measures.

Variable intra‐examiner and inter‐examiner reliability of USI has been reported in the literature [34, 35]. Given the general perception that ultrasonography is a highly operator‐dependent technique [34, 36], our results are encouraging and represent an important step in support of further application of USI to assess other foot joints. The results of this study will inform the methodological development of an USI atlas for grading the degree of osteoarthritic change at the first MTPJ. It is expected that the accompaniment of an illustrated manual (i.e., USI atlas), that clearly presents the USI procedure, features and grades will improve the consistency of interpretation and grading and improve the reliability when examining both structural and inflammatory change in first MTPJ OA.

This study must be viewed in the context of possible limitations. First, the USI procedure developed included only a dorsal scan of the first MTPJ. USI offers a multiplanar technique and as the medial and/or plantar aspect of the first MTPJ was not examined, the developed procedure may underestimate the prevalence or severity of features.

Second, the poor reliability reported for examining cartilage as a continuous measure could be mitigated by segmenting the entire cross‐sectional area of the articular cartilage using ImageJ software [37]. With this technique, the average cartilage thickness within standardized regions can be calculated. However, this technique requires additional expertize and overall time to complete the segmentation, which may limit the translation of cartilage thickness assessment to a clinical setting. Third, investigator bias may have occurred as the same radiologist reported on radiographic screening and graded the acquired ultrasound images. All participants were randomly assigned an alphanumeric code upon entry into the study to minimize this risk of bias. Finally, despite efforts to proactively recruit an ethnically diverse population that represents the broader New Zealand population, no Pacific people were included. Pacific people suffer from significant and longstanding health inequalities and poorer health outcomes compared to the other New Zealanders. Therefore, the collection of accurate ethnic OA data is needed to better understand what factors contribute to these inequalities and to provide the capacity to measure progress.

## 5 CONCLUSION

Our developed USI acquisition procedure and grading system are reliable in evaluating first MTPJ OA features in participants with radiologically confirmed OA. The USI procedure demonstrated good to excellent intra‐examiner reliability in examining all features, except cartilage thickness, when evaluated as a continuous measure. Inter‐examiner reliability was good for grading joint effusion and excellent for grading all other USI features. With all inflammatory features reporting good to excellent intra‐examiner and inter‐examiner reliability, coupled with their prognostic value for structural progression, USI affords an opportunity to detect prognostic markers of OA earlier in the disease cascade. The results of this study will be incorporated into the methodological development of a USI atlas for grading the extent of osteoarthritic change in the first MTPJ.

## AUTHOR CONTRIBUTIONS

**Prue Molyneux**: Conceptualization; formal analysis; funding acquisition; investigation; methodology; project administration; resources; visualization; writing ‐ original draft preparation; writing ‐ review and editing. **Matthew Carroll**: Conceptualization; funding acquisition; methodology; supervision; visualization; writing ‐ review and editing. **Catherine Bowen**: Conceptualization; funding acquisition; methodology; supervision; writing ‐ review and editing. **Richard Ellis**: Conceptualization; methodology; supervision; writing ‐ review and editing. **Keith Rome**: Conceptualization; methodology; supervision; writing ‐ review and editing. **Kate Fitzgerald**: Investigation; formal analysis; software; writing ‐ review and editing. **Phillip Clark**: Investigation; formal analysis; software; writing ‐ review and editing.

## CONFLICT OF INTEREST STATEMENT

Professors Catherine Bowen and Keith Rome are the Editors in Chief UK of the Journal of Foot and Ankle Research. It is journal policy that editors are removed from the peer review and editorial decision‐making processes for papers they have co‐authored. Matthew Carroll is an Editorial Board member of the Journal of Foot and Ankle Research. The remaining authors declare no conflicts of interest in relation to this work.

## ETHICS STATEMENT

This study has been approved by an independent group of people called a Health and Disability Ethics Committee (HDEC), who check that studies meet established ethical standards. The Southern Health and Disability Ethics Committee has approved this study. HDEC Ethics Reference: 2022 FULL 12721.

## CONSENT FOR PUBLICATION

Not applicable.

## Supporting information

Supporting Information S1

Supporting Information S2

## Data Availability

Reasonable requests for data beyond that contained in the Supporting Information will be considered by the corresponding author.
