# Supplementary material for: Reliability of an ultrasound imaging acquisition procedure for examining osteoarthritis in the first metatarsophalangeal joint
Source: J Foot Ankle Res. 2024 Mar 29;17(1):e12002. doi: 10.1002/jfa2.12002 (PMC11296711; doi:10.1002/jfa2.12002)
Supplement: Supplementary file 1 — Supporting Information S1 [file JFA2-17-e12002-s002.docx]

| **Additional file 1: EULAR US reporting recommendations** | | |  |
| --- | --- | --- | --- |
| **Recommendations checklist for reporting studies using ultrasound in rheumatic and musculoskeletal diseases** | | |  |
| Topic | Number | Item to report | Location where item is reported |
| Objective | 1 | Objective of the ultrasound measurement in the study (eg, description, prediction, diagnosis, validation…) | Page: 5  Lines: 128-131 |
| Design | 2 | Study design (eg, cross- sectional, case- control, cohort, randomised clinical trial, …) | Page: 6  Lines: 134-144 |
|  | 3 | Prospective or retrospective data collection* | Pages: 7-10  Lines: 165-244 |
| 150-Participants | 4 | Informed consent procedure (written, oral) | Page: 7  Lines: 162-163 |
|  | 5 | Source, selection criteria and sampling of the participants (including controls where appropriate) | Page: 7  Lines: 151-156 |
| Blinding | 6 | Procedures for blinding of sonographers and participants | Page: 9  Lines 223-224 |
| Ultrasound features | 7 | a. Broad domain* of interest (eg, inflammation or structural damage)  b. Target domain* with corresponding theoretical ultrasound definition(s)* (eg, synovitis: synovial hypertrophy plus increased synovial blood flow)  c. Domain components (ie, elementary lesions)* with corresponding operational definitions* (eg, synovial hypertrophy: increased thickness of synovium with hypoechoic appearance) | Pages: 8-9  Lines: 190-202  Figure 2 |
| Scanning/acquisition procedures | 8 | a. Anatomical region(s)* or structure(s)* that were studied  b. Rationale for choosing these anatomical region(s)/structure(s) | Pages: 5  Lines: 102-111 |
|  | 9 | a. Patient position (eg, prone, supine…)  b. Anatomical region position (eg, neutral…)  c. Surfaces scanned (eg, volar, dorsal)  d. Transducer position (eg, transverse, longitudinal) e. Whether the examination was dynamic* | Page: 9  Lines: 204-217  Figure 3 |
| Ultrasound scoring system | 10 | Scoring system used:  a. Type (eg, quantitative, semiquantitative, binary)  b. Level: (eg, patient level, joint/anatomical region level) | Page: 8  Lines: 192-202  Additional file 2 |
|  | 11 | For existing scoring systems:  a. References or results of previous validity and reliability studies  b. Score range (minimum- maximum), and meaning of the score (eg, higher is ……)  c. Rationale for any thresholds or cut- offs  d. Training session details if performed e. The reliability* of the scoring system in the hands of the study sonographers/readers | NA |
|  | 12 | For new scoring systems:  a. Rationale for developing a new scoring system  b. Detailed description of the scoring system  c. Reliability assessment:  I. Type of reliability: inter- reader, other  ii. Training session if performed  iii. The reliability of the scoring system as applied by the study sonographers/readers  iv. Whether reliability was assessed on static images, video- clips or real- time examination of patients  v. Sample size of the reliability study  vi. Reliability results (eg, kappa or ICC with 95% CI and type of kappa or ICC, prevalence of observed lesions, smallest detectable change, SE of measurement) | Page: 5  a) Lines: 113-125  b) Lines: 192-202 & Additional file 2  c) i. Page: 10, Lines: 250-256  ii. Page: 6, Lines: 137-142  iii. Pages: 10-11, Lines: 250-256  iv. Page 9, Lines: 219-224  v. Page 10, Lines: 238-240 & Figure 1  vi. Page 11, Lines 258-268 |
| Sonographer(s)/reader(s) | 13 | a. Whether acquisition and reading were performed at the same time  b. Whether acquisition and reading were performed by the same person  c. Number of sonographers or readers  d. In longitudinal studies, whether the same sonographer scanned the same patient at each assessment | Pages: 9-10  Lines: 219-244 |
|  | 14 | Optional: Information about the experience of sonographer(s) and reader(s) (eg, numbers of scanned performed, certification, qualification…) | Page: 9  Lines: 219-222 |
| Equipment | 15 | a. Brand and model of the ultrasound device  b. Type and model of the transducer  c. Whether the ultrasound device (or software) was changed during the study | Page: 10  Lines: 226-235 |
|  | 16 | Ultrasound modalities* and settings  a. Grey scale  b. Doppler  c. Other | Page: 10  Lines: 229-235 |
| Images (pictures and drawings) | 17 | For images included into the manuscript, verify that:  a. Information identifying patient is deleted  b. Essential targets in the image(s) are clearly labelled  c. Images match the content of the manuscript  d. Quality of the images is adequate | No images that could identify a participant are included in the manuscript |
| Contextual factors | 18 | Duration of ultrasound examination when relevant for the study question | NA |
|  | 19 | *Optional:*  *a. Whether ambient conditions (eg, temperature, time of day) were kept stable during the study*  *b. Potential confounding factors (eg, exercise, alcohol, caffeine, smoking)* | NA |
| Statistical analysis | 20 | a. Existence of a pre- specified statistical analysis plan and specification of post- hoc analyses  b. Analyses performed  c. Whether the analyses were performed at patient or at joint/region level  d. Extent of missing data  e. Handling of missing data | Pages: 10-11  Lines 246- 256 |
| Disclosures | 21 | Potential conflicts of interest including those related to ultrasound | Pages: 16-17  Lines: 391-425 |
