# Supplementary material for: Reliability of an ultrasound imaging acquisition procedure for examining osteoarthritis in the first metatarsophalangeal joint
Source: J Foot Ankle Res. 2024 Mar 29;17(1):e12002. doi: 10.1002/jfa2.12002 (PMC11296711; doi:10.1002/jfa2.12002)
Supplement: Supplementary file 2 — Supporting Information S2 [file JFA2-17-e12002-s001.docx]

## Additional file 2

# USI grading system

| **USI feature** | **USI acquisition procedure** | | **Grading system** | | | |  |
| --- | --- | --- | --- | --- | --- | --- | --- |
| **Joint Effusion** | Dorsal | Longitudinal | 0 | 1 | 2 | 3 |  |
|  |  | Transverse | 0 | 1 | 2 | 3 |  |
| **Synovial Hypertrophy** | Dorsal | Longitudinal | 0 | 1 | 2 | 3 |  |
|  |  | Transverse | 0 | 1 | 2 | 3 |  |
| **Synovitis** | Dorsal | Longitudinal with power Doppler | 0 | 1 | 2 | 3 |  |
| **Joint Space Narrowing** | Dorsal | Longitudinal – scan right through joint | 0 | 1 | 2 | 3 | _mm_ |
| **Osteophytes** | Dorsal | Longitudinal – scan right through joint | 0 | 1 | 2 | 3 | _mm_ |
|  |  | Transverse | 0 | 1 | 2 | 3 | _mm_ |
| **Cartilage** | Dorsal | Longitudinal – scan right through joint | 0 | 1 | 2 | X | _mm_ |

**Scoring**

1. **= Absent**
2. **= Mild**
3. **= Moderate**
4. **= Severe**
